# ASO Author Reflections: PARP7 Association to Estrogen and Immunity in Clinical Breast Cancer

**DOI:** 10.1245/s10434-026-19296-8

**Published:** 2026-02-21

**Authors:** Anelia Kassi, Luke Rodriguez, Rongrong Wu, Takashi Ishikawa, Kazuaki Takabe

**Affiliations:** 1 Department of Surgical Oncology, Roswell Park Cancer Institute, Buffalo, NY USA; 2 Department of Breast Surgery and Oncology, Tokyo Medical University, Tokyo, Japan; 3 Department of Surgery, University at Buffalo Jacob School of Medicine and Biomedical Sciences, the State University of New York, Buffalo, NY USA; 4 Department of Breast Surgery, Fukushima Medical University, Fukushima, Japan; 5 Department of Surgery, Niigata University Graduate School of Medical and Dental Sciences, Niigata, Japan; 6 Department of Gastroenterological Surgery, Yokohama City University Graduate School of Medicine, Yokohama, Japan

## Past

Poly (ADP-ribose) polymerases (PARPs) are a family of 17 enzymes involved in several essential cellular processes, including DNA repair.^1^ This property was exploited for the development of targeted therapy that inhibits PARP, which causes synthetic lethality of homologous recombination repair deficient neoplasms, notably *BRCA*-mutated breast, ovarian, prostate, and pancreatic cancers.^2^ However, only a subset of PARP (PARP 1, 2, and 3) participate in the repair of DNA.^2^ The functions of other PARPs are varied, but their implications are not well established. PARP7 is implicated in cellular stress signaling, gene regulation, and innate immunity in breast and prostate cancer, but its exact oncologic role remains unclear.^3^ In the preclinical setting, PARP7 blocks interferon type 1 (IFN-1) signaling and ultimately dampens antitumor immunity, while its inhibition enhances immune activity.^1^ Others have reported that PARP7-dependent repression of the estrogen receptor (ER) alpha occurs upon estradiol binding in luminal A breast cancer and decreases cancer cell proliferation in preclinical studies.^3^ We sought to investigate the clinical relevance of PARP7 gene expression in large cohorts of patients with breast cancer linked with transcriptomes.

## Present

We conducted a large transcriptomic study of 6063 patients with breast cancer using the Cancer Genome Atlas (TCGA, *n* = 1090), the Molecular Taxonomy of Breast Cancer International Consortium (METABRIC, *n* = 1904), and The Sweden Cancerome Analysis Network-Breast (SCAN-B, GSE96058, *n* = 3069). Clinicopathological parameters were obtained from these databases, and a comprehensive gene expression profile analysis was performed to compare patients with high versus low PARP7 levels.

Patients with high PARP7 expression had significantly better overall survival in two of three cohorts. ER-positive/HER2-negative breast cancers had higher levels of PARP7 than the other subtypes. High PARP7 expression was associated with high estrogen and androgen response consistently in all cohorts. High PARP7 levels were associated with higher IFN-1 but lower IFN-beta1 and CXCL10, and lower immune cell infiltration, but did not affect overall cytolytic activity. High PARP7 expression was associated with lower cell proliferation shown consistently by Ki67, histological grade, proliferation score, and Gene Set Enrichment Analyses. Taken together, we found that high PARP7 expression in patients with breast cancer is not only associated with estrogen and androgen response but also with lower cell proliferation and better overall survival.^4^

## Future

Our study inquired upon the clinical relevance of PARP7 gene expression in patients with breast cancer. We found that high PARP7 levels were associated with enhanced estrogen response, less cell proliferation, and better overall survival. Unlike previous reports using preclinical data, high PARP7 levels did not ultimately affect cytolytic activity despite increased immune cell infiltration or IFN-1 in patients. Multiple PARP7 inhibitors have been investigated in preclinical cellular and xenograft models.^1,5^ Alone or in combination with other drugs, they have demonstrated tumor growth inhibition up to 83%, by restoring IFN-1 activity.^5^ The phase 1 trial of a selective PARP7 inhibitor RBN-2397 showed promising results in advanced solid tumors, including breast and PARP7-amplified cancers (NCT04053673).^1,5^ Improved understanding of PARP7 and other members of the PARP family using real-world data may continue to expand targeted-therapy options in breast cancer.

## References

[1] Gozgit JM, Vasbinder MM, Abo RP, et al. PARP7 negatively regulates the type I interferon response in cancer cells and its inhibition triggers antitumor immunity. *Cancer Cell*. 2021;39(9):1214-1226.e1210. 34375612 10.1016/j.ccell.2021.06.018

[2] Rose M, Burgess JT, O’Byrne K, Richard DJ, Bolderson E. PARP inhibitors: clinical relevance, mechanisms of action and tumor resistance. *Front Cell Dev Biol*. 2020;8:564601. 33015058 10.3389/fcell.2020.564601 PMC7509090

[3] Rasmussen M, Tan S, Somisetty VS, et al. PARP7 and mono-ADP-ribosylation negatively regulate estrogen receptor α signaling in human breast cancer cells. *Cells*. 2021;10(3):623. 33799807 10.3390/cells10030623 PMC8001409

[4] Rodriguez L, Wu R, Kassi A, et al. High PARP7 expression is associated with higher estrogen response and immune suppression, but less cell proliferation and better survival in breast cancer. *Ann Surg Oncol*. 2026. 10.1245/s10434-026-19133-y. 41712159 10.1245/s10434-026-19133-y

[5] Popova K, Benedum J, Engl M, et al. PARP7 as a new target for activating anti-tumor immunity in cancer. *EMBO Mol Med*. 2025;17(5):872–88. 40128585 10.1038/s44321-025-00214-6 PMC12081928

